# Oral flora of stray dogs and cats in Algeria: *Pasteurella* and other zoonotic bacteria

**DOI:** 10.14202/vetworld.2020.2806-2814

**Published:** 2020-12-30

**Authors:** Kahina Razali, Rachid Kaidi, Amine Abdelli, Mohamed Nabil Menoueri, Khatima Ait-Oudhia

**Affiliations:** 1 Laboratory of Animal Reproduction Biotechnologies, Blida, Algeria; 2 Department of Veterinary Sciences, Institute of Veterinary Sciences, Université Saad Dahlab de Blida 1, Blida, Algeria; 3 Department of Agriculture Science, Bouira University, Bouira, Algeria

**Keywords:** Algeria, bite, dogs and cats, oral flora, *Pasteurella*

## Abstract

**Background and Aim:**

Knowledge of potentially pathogenic bacteria presents in the oral cavity of dogs and cats may be helpful in determining appropriate treatment for infected bite wounds. About 120.000 people are exposed to dog and cat bites every year in Algeria, but little is known about the dog and cat oral flora causing bite wound complications. The purpose of this study was to identify potential zoonotic bacteria from oral cavity of dogs and cats and to determine their susceptibility to antibiotics to contribute to the treatment of bite wound infection.

**Materials and Methods:**

Oral swabs from 100 stray dogs and 100 stray cats were collected and cultured in several media: Chocolate agar, MacConkey agar, and Mannitol Salt Agar. Bacterial isolates were identified using several commercial kits of the analytical profile index and tested for antibiotic susceptibility by disk diffusion method.

**Results:**

Overall, 185/200 (92.5%) dogs and cats carried zoonotic bacteria in their mouths, of which 55.13% (102/185) had at least two bacterial pathogens. 374 pathogenic strains belonging to 15 genera were isolated: Eleven were Gram-negative (*Proteus*, *Pasteurella*, *Escherichia*, *Moraxella*, *Klebsiella*, *Acinetobacter, Enterobacter, Pseudomonas*, *Aeromonas*, and *Neisseria* *Haemophilus*) and four were Gram-positive (*Staphylococcus*, *Streptococcus*, and *Corynebacterium*, *Bacillus*). Fifty-one strains of *Pasteurella* were isolated from 44 carriers of *Pasteurella* (21 *Pasteurella multocida*, 21 *Pasteurella pneumotropica*, and 9 *Pasteurella* spp.). *Pasteurella* strains were tested for antibiotic resistance. Resistance to at least one drug was observed in 8 (15.68%) of *Pasteurella* isolates and two strains (3.92%) were found to be multidrug-resistant (to two or more drugs). Erythromycin, penicillin, and ampicillin were the antimicrobials to which the isolates showed greater resistance (7.84%, 5.88%, and 3.92%, respectively).

**Conclusion:**

To the best of our knowledge, this study is the first in Algeria to detect potential human pathogenic bacteria in the oral cavity of dogs and cats. It reveals that these animals have multiple zoonotic bacteria in their mouths including *Pasteurella* species, which may be multidrug-resistant.

## Introduction

Animal bites are a major health problem worldwide [1,2], due to the high number of people seeking health services for animal-related injuries [2,3]. Dogs and cats are implicated in 90% of this and account for approximately 1% of the annual emergency department visits [3-7]. The most common bite-related complication is wound infection [6,7], resulting in local and systemic infections requiring specific antimicrobial therapy [7-9]. It is estimated that approximately 3-18% of dog bites and 20-80% of cat bites become infected [5-7] by the oral flora of the biting animal [10,11]; wounds are usually polymicrobial [4-10,11] and contain a mixture of aerobic and anaerobic bacteria [2,3].

Although oral flora of dogs and cats may contain several zoonotic pathogens [2-12], *Pasteurella* species, in particular *Pasteurella multocida*, has been reported as one of the major bacteria leading to human infection following animal bites [5]. Thus, with up to 66% of dogs and 90% of cats harboring *Pasteurella* species in their mouths [13], the number of bites inoculate this microorganism is significant. In humans, *P*. *multocida* is isolated from 50% of dog bite wounds, 75% of cat bite wounds or scratches, and less frequently from licks [13,14].

Typical clinical manifestations are cellulitis, soft-tissue abscesses, and purulent wounds at the site of injury; the infection is serious and can be complicated in tenosynovitis, osteomyelitis, and septic arthritis [9-14]. In addition to local wound infection, *P. multocida* can cause systemic infections, including septicemia, meningitis, brain abscess, pneumonia, endocarditis, and other severe sequelae especially in immunocompromised patients [9-13].

In Algeria, the number of stray dogs and cats wandering freely through the streets and urban areas is constantly increasing over the last few years, placing people at risk of bites [15,16].

Furthermore, around 120.000 people are exposed to animal bites each year, 80% of whom are bitten by stray dogs [15,16].

Knowledge of zoonotic bacteria present in the oral cavity of dogs and cats is very important for determining the danger of bite complications in humans [4-11], and the determination of their antibiotic susceptibility helps doctors to select the appropriate treatment for infected bite wounds [2-14].

Despite the high frequency of annual visits to the emergency departments, in our country, due to animal bites [15,16], the oral flora of dogs and cats causing bite wound complications is still relatively unstudied.

The purpose of this study was to identify potential zoonotic bacteria from oral cavity of dogs and cats and to determine their susceptibility to antibiotics to contribute to the treatment of bite wound infection.

## Materials and Methods

### Ethical approval

The study protocol was approved by the ethics committee and decision board (number 01/2018) of P.I.C.C-U.H.E.P. of Algiers.

### Study area

The study was conducted in the Department of Algies which is situated on the central coast of Algeria between 3°2’31.09’’ east longitude and 36°45’9’’ north latitude. It covers an area of 1190 km^2^ and comprises 57 districts with a population of over 2.9 million inhabitants. Algiers is bounded from the north by the Mediterranean Sea, from the south by the Blida department, from the east by the Boumerdes department, and from the west by the Tipaza department. Public Industrial and Commercial Company-Urban Hygiene and Environmental Protection (P.I.C.C-U.H.E.P) is affiliated with the Algerian Ministry of Water Resources and Environment that controls zoonosis and vector-borne diseases such as rabies and leishmaniosis.

**Figure-1:**
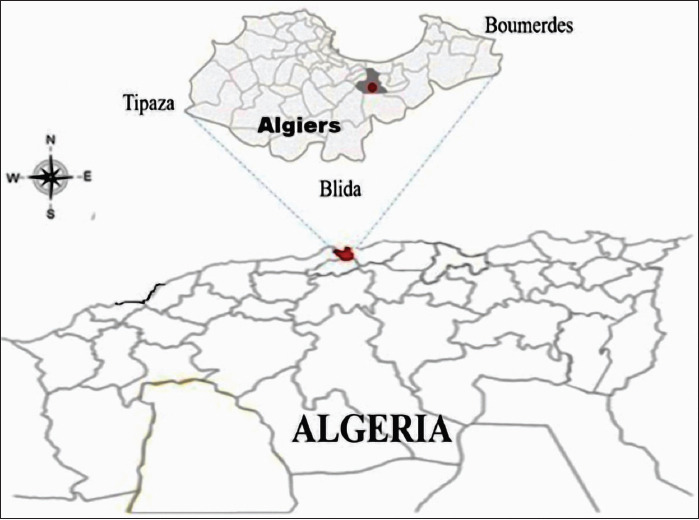
Map of Northern Algeria showing the location of the study area in Algiers [Source: Map prepared by the corresponding author].

In the context of the National Rabies Prevention Program, P.I.C.C-U.H.E.P. Catches stray dogs and cats in the 57 districts of the Algiers department. The captured animals are subsequently sheltered in the dog-pound of El-Harrach during the legal period (7 days) before euthanasia to allow for owners to claim their pets in compliance with the Algerian legislation on the protection of animals. The geographical position of the dog-pound and the animal catching radius in Algiers is represented in Figure-1.

### Sampling

From January 2018 to July 2019, 200 buccal swabs (tongue, gum, palate, cheeks, and teeth) were collected at irregular intervals from 100 stray dogs and 100 stray cats which were randomly selected without distinction of age, sex, or breed. All the sampled ­animals were caught by the P.I.C.C-U.H.E.P. from the 57 municipalities of Algiers during the study period that was apparently healthy and did not receive any previous treatment.

### Bacterial analysis of samples

The specimens were transported in an icebox (+4°C) within 60 min after collection to the microbiology laboratory for further analysis. Following a control quality step, *Escherichia coli* American Type Culture Collection (ATCC) 25922, *Staphylococcus aureus* resistant Type Culture Collection (ATCC) 43300, *S. aureus* sensitive Type Culture Collection (ATCC) 25923, and *Pseudomonas aeruginosa* Type Culture Collection (ATCC) 27853 were used.

Each swab was directly plated on chocolate agar (Columbia agar medium supplemented with 5% of blood sheep and slowly heating to 80°C) (Pasteur Institute, Algeria), MacConkey agar (Pasteur institute, Algeria), and Mannitol salt agar “Chapman Medium” (Pasteur institute, Algeria). Chocolate agar plates were incubated for 48-72 h at 37°C in a 5% CO_2_ atmospheric chamber, and both MacConkey and Mannitol salt agar plates were incubated aerobically at 37°C for 24 h.

After 24, 48, and 72 h of incubation, the plates were examined and different types of colonies were then subcultured separately to the appropriate media for the processing of pure cultures. When pure cultures were obtained, each isolate was identified according to the morphology of the colonies, the type of hemolysis, the Gram staining microscopy, and several biochemical tests for characterizing isolates.

Biochemical tests included: Catalase and oxidase activities, substrate utilization as determined by commercial biochemical test kits, and coagulase testing using rabbit plasma for Staphylococci identification. *Pasteurella* spp. were identified on the basis of Gram staining (Gram-negative coccobacilli), absence of hemolysis, absence of growth on MacConkey, and positive biochemical reactions: Catalase and oxidase activities, and indole test. Final identification of strains was done using API20NE commercial kit (Biomérieux, Marcy l’Etoile, France).

Several commercial analytical profile index (API) kits (API20NE, API20E, APINH, API Staph, API Strept) (Biomérieux, Marcy l’Etoile, France) were used to identify strains other than *Pasteurella*.

### Antimicrobial susceptibility testing of *Pasteurella*

A subset of *Pasteurella* strains were tested for susceptibility to four antibiotics which are commonly prescribed for the treatment of animal bites. Resistance to amoxicillin/clavulanic acid 30 μg; penicillin 10 IU; ampicillin 10 μg; tetracycline 30 μg; and erythromycin 5 μg was determined on Muller-Hinton agar supplemented with 5% sheep blood (Pasteur Institute, Algeria) using the standard Kirby–Bauer disk diffusion method.

### Statistical analysis and map conception

Pearson’s Chi-square test (α=5%) was applied to compare *Pasteurella* prevalence, using Microsoft Excel world 2007. Differences were statistically significant when p<0.05.

The map showing the geographic location of the dog-pound and the animal catching area was conceived using Microsoft PowerPoint 2007, as available in Microsoft Office software (Microsoft, USA), while the quality of the artwork was enhanced by Adob Photoshop software.7.0.1 (Adobe, USA).

## Results

### Prevalence and bacterial isolation

During the study period, a total of 100 stray dogs and 100 stray cats were swabbed. Bacteriological culture showed that 92.5% (185/200) of the swabbed animals had at least one zoonotic bacterium in their mouths. The carrier rates for zoonotic bacteria were similar in dogs (92%, 92/100) and cats (93%, 93/100). From 185 carriers of zoonotic bacteria, 374 pathogenic strains were isolated (174 and 200 in dogs and cats, respectively) (Table-1).

**Table-1:** Prevalence of oral zoonotic bacteria in stray dogs and cats in Algeria.

Characteristics	Total		Dog		Cat	
		
	No.	%	No.	%	No.	%
Sampled animals’	200	-	100	-	100	-
Carriers of zoonotic bacteria	185	92.5	92	92	93	93
Zoonotic bacterial isolates	374	-	174	-	200	-

Potential zoonotic bacterial isolates accounted for 62.83% (63.22% in dogs and 62.5% in cats) Gram-negative and 37.17% (36.78% in dogs and 37.5% in cats) Gram-positive which were mainly represented by cocci (25.67%), including 16.58% of coagulase-positive Staphylococci and rods (11.5%) representing *Corynebacterium* spp. and *Bacillus* spp.

From the Gram-negative standpoint, 43.05% of the bacteria were rods; 18.98% were coccobacilli, and 0.8% were cocci.

The most common Gram-negative bacteria isolates were enterobacteria (25.13%, 94/374), non-fermenting Gram-negative bacilli (15.77%, 59/374), and *Pasteurella* (13.63%, 51/374). *Proteus mirabilis* was the most frequently encountered (14.97%, 56/374) of enterobacteria isolates, followed by *E. coli* (7.22%, 27/374).

Isolation rates of pathogenic Gram-negative bacteria were similar for dogs (63.22%, 110/174) and cats (62.50%, 125/200), in this study, isolation rates of pathogenic Gram-positive bacteria as well (36.78%, 64/174 dogs and 37.5%, 75/200 cats). Among Gram-negative bacteria, *Klebsiella oxytoca* was isolated only from the oral cavity of the cat (1.87%, 7/200), while *Aeromonas hydrophila* was detected only in the oral cavity of the dog (2.3%, 4/174). Detection rates of *Moraxella* and *Acinetobacter* were lower for dogs (1.72%, 3/174 and 0.57%, 1/174, respectively) compared to cats (4.5%, 9/200; and 0.57%, 1/174, respectively) (Table-2).

**Table-2:** Zoonotic bacteria isolated from oral cavities of stray dogs and cats in Algeria and their frequency.

Zoonotic bacterial isolates Microscopic shape/species	Total		Dog		Cat	
		
	No.	%	No.	%	No.	%
Total isolates	374	-	174	-	200	-
Gram-positive bacteria	139	37.17	64	36.78	75	37.5
Cocci	96	25.67	45	25.86	51	25.5
CoPS	62	16.58	34	19.54	28	14
*Streptococcus* spp.	34	9.09	11	6.32	23	11.5
Rods	43	11.5	19	10.92	24	12
*Corynebacterium* spp.	31	8.29	12	6.9	19	9.5
*Bacillus* spp.	12	3.21	7	4.02	5	2.5
Gram negative bacteria	235	62.83	110	63.22	125	62.5
Cocci	3	0.8	1	0.57	2	1
*Neisseria* spp.	3	0.8	1	0.57	2	1
Coccobacilli	71	18.98	36	20.69	35	17.5
*Acinetobacter baumannii*	6	1.6	1	0.57	5	2.5
*Pasteurella multocida*	21	5.61	10	5.75	11	5.5
*Pasteurella pneumotropica*	21	5.61	17	9.77	4	2
*Pasteurella* spp.	9	2.41	4	2.3	5	2.5
*Haemophilus influenza*	2	0.53	1	0.57	1	0.5
*Moraxella* spp.	12	3.21	3	1.72	9	4.5
Rods	161	43.05	73	41.95	88	44
*Escherichia coli*	27	7.22	11	6.32	16	8
*Klebsiella oxytoca*	7	1.87	-	-	7	3.5
*Proteus mirabilis*	56	14.97	29	16.67	27	13.5
*Enterobacter cloacae*	4	1.07	1	0.57	3	1.5
*Pseudomonas aeruginosa*	4	1,07	2	1.15	2	1
*Aeromonas hydrophila*	4	1.07	4	2.30	-	-
Other NFGNB	59	15.78	26	14.94	33	16.5

CoPS=Coagulase positive *Staphylococcus*, NFGNB=Non-fermenting Gram-negative bacilli

Out of the 200 bacteriologically cultured swabs, 185 showed at least one type of colony in which 374 zoonotic bacterial species were isolated after identification. Eighty-three (44.86%) animals, including 44 dogs and 39 cats carried only one bacterial species, while the remaining 102 animals (48 dogs and 54 cats) carried at least two different bacterial species within a maximum of eight bacterial species for dogs and six bacterial species for cats (Table-3).

**Table-3:** Number of zoonotic bacteria isolated per animal.

No of carried zoonotic bacteria	Total population		Dog		Cat	
		
	No	%	No	%	No	%
Total animals	185	-	92	-	93	-
1 bacteria	83	44.86	44	47.82	39	41.93
2 bacteria	51	27.56	29	31.52	22	23.65
3 bacteria	30	16.21	11	11.95	19	20.43
4 bacteria	13	7.027	5	5.43	8	8.6
5 bacteria	3	1.62	1	1.08	2	2.15
6 bacteria	4	2.16	1	1.08	3	3.22
8 bacteria	1	0.54	1	1.08	-	-

### *Pasteurella* spp. carriage descriptive data

Of the 200 dogs and cats examined during the study period, 44 (22%) carried *Pasteurella* spp. in their oral cavities; the carrying rates for dogs and cats were 27% (27/100) and 17% (17/100), respectively. Fifty-one *Pasteurella* strains were isolated and distributed as follows: 38 animals carried one *Pasteurella* strain; five animals carried two *Pasteurella* strains and one cat carried three *Pasteurella* strains. Identification of the isolates revealed that 21 of the strains were *P. multocida*, 21 strains *Pasteurella pneumotropica*, and nine strains (*Pasteurella* spp.) remained unclassified (Tables-4 and 5).

**Table-4:** Isolation rates of *Pasteurella* spp. from oral cavities of stray dogs and cats in Algeria.

Characteristics	Total		Dog		Cat	
		
	No.	%	No.	%	No.	%
Samled animals’	200	100	100	100	100	100
No. of subjects	44	22	27	27	17	17
No. of isolates	51	100	31	100	20	100
*Pasteurella multocida*	21	41.17	10	32.25	11	55
*Pasteurella pneumotropica*	21	21.56	17	54.83	4	20
*Pasteurella* spp.	9	9.8	4	12.9	5	25

**Table-5:** *Pasteurella* species isolated from oral cavities of stray dogs and cats in Algeria and their frequency.

Characteristics	Total population		Dog		Cat	
		
	No	%	No	%	No	%
Carriers of *Pasteurella*	44	-	27	-	17	-
1 species	38	86.36	23	85.18	15	88.23
*P. pneumotropica*	16	36.36	13	48,15	3	17,65
*P. multocida*	16	36.36	7	25,93	9	52,94
*Pasteurella* spp.	6	13.64	3	11,11	3	17,65
2 species	5	11.36	4	14.81	1	5.88
*P. multocida+ Pasteurella* spp.	1	-	-	-	1	5.88
*P. multocida+P. pneumotropica*	3	6.82	3	11.11	-	-
*Pasteurella* spp.*+P. pneumotropica*	1	2.27	1	3.70	-	-
3 species	1	2.27	-	-	1	5.88
*P. multocida+Pasteurella* spp.*+P. pneumotropica*	1	2.27	-	-	1	5.88

P. multocida=Pasteurella multocida, P. pneumotropica=Pasteurella pneumotropica

There were some differences in the distribution of *Pasteurella* species between dogs and cats: *P. multocida* was the predominant species in cats (55% of feline isolates), while *P. pneumotropica* was the predominant species in dogs (55% of canine isolates).

In fact, there was no difference between dogs (10%) and cats (11%) in carrying *P. multocida*, but there was a difference in carrying *P. pneumotropica*: Of the 100 dogs sampled, 17 (17%) carried *P. pneumotropica*, although it was only found in four cats (4%) (Table-4).

### Susceptibility to antibiotics

Antibiotic susceptibility results showed that 18 (69.23%) isolates were susceptible to all antimicrobial drugs tested, while 30.67% of the strains were resistant to one or two antibiotics. Of the eight antibiotic-resistant isolates, five distinct antibiotic-resistant patterns were observed: Bi-drug-resistance was observed in *P. pneumotropica* carried by a dog, while tri-drug resistance was observed in *P. multocida* isolated from a cat (Tables-6 and 7).

**Table-6:** Antibiotic susceptibility of *Pasteurella* spp. isolated from oral cavities of stray dogs and cats in Algeria.

*Pasteurella* isolates	Total (n=26)		Isolates from dog (n=17)		Isolates from cat (n=9)	
			
Antimicrobial agents	R (%)	S (%)	R (%)	S (%)	R (%)	S (%)
Penicillin	3 (11.53)	23 (88.47)	2 (11.76)	15 (88.24)	1 (11.11)	8 (88.89)
Ampicillin	2 (7.69)	24 (92.31)	1 (5.88)	16 (94.12)	1 (11.11)	8 (95.0)
Erythromycin	4 (15.38)	22 (84.62)	2 (11.76)	15 (88.24)	2 (22.22)	7 (77.78)
Tetracycline	1 (3.84)	25 (96.16)	1 (5.88)	16 (94.12)	0 (0.0)	9 (100)
Amoxicillin-Clavulanate	1 (3.84)	25 (96.16)	1 (5.88)	16 (94.12)	0 (0.0)	9 (100)

R=Resistant, S=Susceptible

**Table-7:** Antibiotic resistance patterns in *Pasteurella* spp. isolated from oral cavities of stray dogs and cats in Algeria.

Patterns/isolates	Total	*Pasteurella multocida*		*Pasteurella pneumotropica*		*Pasteurella* spp.	
		
		Dog	Cat	Dog	Cat	Dog	Cat
No. Isolates	26	5	6	11	1	1	2
S^ble^ (%)	18 (69.23)	3 (60)	4 (66.66)	7 (63.63)	1 (100)	1 (100)	2 (100)
Tetracycline	1 (3.84)	1 (20)	-	-	-	-	-
Amox-clav	1 (3.84)	-	-	1 (9.09)	-	-	-
Ery	3 (11.53)	1 (20)	1 (16.66)	1 (9.09)	-	-	-
Penicillin	1 (3.84)	-	-	1 (9.09)	-	-	-
Pen-Amp	1 (3.84)	-	-	1 (9.09)	-	-	-
Pen-Amp-Ery	1 (3.84)	-	1 (16.66)	-	-	-	-

From the effectiveness therapy point of view, erythromycin, penicillin, and ampicillin were antimicrobials for which isolates showed higher resistance rates with 7.84%, 5.88%, and 3.92% of isolates, respectively (Table-6).

## Discussion

Human infected-wounds caused by dog and cat bites are usually polymicrobial, containing a mixture of aerobic and anaerobic bacteria [2,3], mainly through the oral flora of biting animals [5,10,11].

In this study, most of the dogs and cats sampled (92.5% and 185/200) were carriers of zoonotic bacteria in their oral cavities: In 83 animals, a single species was found, 50% of the animals sampled carried two species, and the rest had three or more species. These findings are in accordance with earlier studies [4,11,12,17,18] that reported various opportunistic and potentially pathogenic human bacteria are found in the oral cavity of dog and cat.

Bacteria which are not pathogenic to humans were not included in this work, as well as anaerobic bacteria; 374 bacterial strains were isolated (174 in dogs and 200 in cats), belonging to ten several families: *Staphylococcaceae*, *Streptococcaceae*, *Corynebacteriaceae, Bacillaceae*, *Neisseriaceae*, Pasteurellaceae, *Moraxellaceae*, *Enterobacteriaceae*, *Pseudomonadaceae*, and *Aeromonadaceae*.

Gram-negative bacteria were the most frequently isolated pathogen, with 62.80% of all isolates, while, Gram-positive bacteria represent only 37.20% of the pathogenic bacteria accrued in oral cavities of dogs and cats.

Several studies [19-23] described Gram-negative bacteria as the most common bacteria involved in animal bite complications, such as *Pasteurella* spp., *Neisseria* spp., *Moraxella* spp., and *Enterobacteriaceae*.

It is common knowledge that Gram-negative bacteria are often pathogenic; in addition to, septicemia and bacteremia that contain endotoxins whose release into soft-tissue and blood resulting in acute shock and death [24].

The high proportion of Gram-negative bacteria in dogs’ and cats’ mouths highlights the potential risk of complicating bite wounds infected with these microorganisms. Among Gram-negative bacteria, the predominant family was Enterobacteriaceae, followed by *Pasteurellaceae*, while *Moraxellaceae*, *Pseudomonadaceae*, *Aeromonadaceae*, and *Neisseriaceae* were the less abundant families.

Members of the Enterobacteriaceae family, which includes *E. coli* and *P. mirabilis*, are known to be natural inhabitants of the gastrointestinal tract of humans and animals [20-25]. These bacteria are also found in the esophagus and/or the mouth of dogs and cats [23,25,26].

In this report, the prevalence of *E. coli* in dogs and cats was relatively high (6.32% and 8% oral carriers, respectively) and similar to the 6.12% (dog) and 6.52% (cat) rates reported in South Africa and India, respectively [27,28]. However, *P. mirabilis* isolation rates in our study (16.67% in dogs and 13.5% in cats) appeared to be higher than those reported in South Africa (2.04% in dogs) and India (4.34% in cats) [27,28]. Our abundance of *P. mirabilis* isolation may be attributed to the contamination of food, water, soil, and the environment by fecal flora and licking between animals [25].

Both *E. coli* and *P. mirabilis* are very important human pathogens that lead to numerous health manifestations, such as wound infections and abscesses, urinary tract infection, septicemia, meningitis, and fatal endotoxemia [20-25]. It is also established that these bacteria are multidrug-resistant enterobacteria that generate nosocomial infections [26].

In the current study, *Pasteurella* was the most popular genus (17.81% of all isolates) in dogs, while in cats; it was the second most prevalent genus (10% of all isolates). Several previous studies have described *Pasteurellaceae* (mainly *Pasteurella*) as the most predominant genus in the oral cavities of healthy dogs and cats [4-11-27].

The isolation rate of *Pasteurella* in dogs (17.81%) appeared to be close to the 18.36% reported by Almansa Ruiz *et al*. [28] in South Africa. Whyte *et al*. [12] revealed a lower prevalence of *Pasteurella* (7.52%) in cats. Interestingly, the prevalence of *Pasteurella* in dogs (27%) was higher than that of cats (17%), which conflicts with other reports stating that the main carriers of *Pasteurella* are cats (up to 90%) [4,12,13,17].

This can be due to many factors, such as age, sex, breed, diet, and living conditions [12-29]. In addition, many studies show that the normal oral flora varies depending on the sampling season, sampling mouth area, periodontal health [12-30], and geographical location [29].

*Pasteurella* organisms grow in culture on a variety of commercial media, including sheep blood and chocolate agar [14], but, they are fastidious and can be difficult to isolate and identify from oral flora containing many and various bacteria [29].

In this research, 51 strains of *Pasteurella* were isolated from 44 carriers of *Pasteurella*, including *P. multocida* (21/374, 5.61%), *P. pneumotropica* (21/374, 5.61%), and *Pasteurella* spp. (9/374, 2.41%). Some differences in the distribution of *Pasteurella* species among dogs and cats were observed; *P. multocida* was the most dominant cat species (11/20, and 55% of *Pasteurella* isolates), while *P. pneumotropica* was found to be more abundant in dogs’ mouths (17/31, and 55% of *Pasteurella isolates*). Statistical analysis did not show any significant difference in *P. multocida* prevalence between dogs and cats (p=0.817); however, a significant difference was observed in the prevalence of *P. pneumotropica* (p=0.002).

*Pasteurella* species are small, non-motile, facultative anaerobic, Gram-negative coccobacilli, found in the upper respiratory tracts of many domestic and wild animals, including dogs and cats, which have particularly high colonization rates [13-31].

In humans, *P. multocida* is the most common pathogens isolated from soft-tissue infections resulting from 50% of dog bites, 75% of cat bites or scratches, and less frequently from licks on skin abrasions by pets [9,13,14,31].

*P. multocida* can lead to local wound infection (subcutaneous abscesses, and lymphangitis) may be complicated into cellulitis, arthritis, tenosynovitis, and osteomyelitis [9-4]. In immunocompromised patients, many cases of systemic infections were reported, including septicemia, meningitis, brain abscess, pneumonia, and endocarditis [9-13].

*P. multocida* is most commonly associated with bite infections among members of the *Pasteurella* genus; however, other *Pasteurella* species such as *Pasteurella dagmatis*, *Pasteurella canis*, and *Pasteurella stomatis* found in dogs and cats’ mouths, have also been increasingly cultivated from bite wounds [14-31].

In this study, the isolation rate of *P. pneumotropica* was 17% in dogs and 4% in cats, but *P. dagmatis*, *P. stomatis*, and *P. canis* were not isolated *P. pneumotropica* is known to be more common in laboratory mouse and rodent [32], although, some studies have reported isolation of *P. pneumotropica* from other species, like dogs [33].

Based on the biochemical characteristics, the strains initially identified as *P. pneumotropica* were divided into three biotypes named Jawetz, Heyl, and Henriksen. Both the biotypes Jawetz and Heyl are associated with rodents, while, the biotype Henriksen which was reclassified as *P. dagmatis* was primarily associated with cats and dogs [33].

One of the limitations of this study is that the API20NE commercial kits used to identify *Pasteurella* strains did not include many species of *Pasteurella* other than *P. multocida* and *P. pneumotropica* in their database. Thus, often, *P. dagmatis*, *P. stomatis*, and *P. canis* may often be misidentified as *P. multocida* or *P. pneumotropica* [33].

In addition, Benga *et al*. [33] reported that *P. dagmatis* was formerly designed as *P. pneumotropica* bioptype Henriksen, thus explaining the numerous early reports of clinical diseases caused by *P. pneumotropica* resulting from bites by dogs or cats.

*P. pneumotropica* may occasionally cause disease in both immunodeficient and immunocompetent individuals [32]. Other common Gram-negative bacteria implicated in bite complications were isolated at low frequency, including, *Moraxella* (3.21%), *Acinetobacter* (1.6%), *K. oxytoca* (1.87%), *A. hydrophila* (1.07%), *Enterobacteriaceae* (1.07%), *P. aeruginosa* (1.07%), and *Neisseria* (0.8%).

*Moraxella* and *Neisseria* were reported as part of the normal oral flora of dogs and cats and as an important pathogens in human wounds secondary to animal bites [4], causing severe disease in people including skin and tendon infections, as well as septicemia [9-34].

*A. hydrophila* has been shown to cause serious fatal infections in humans following bite wound infections [20].

In agreement with other investigations, culture-based study of the oral flora of dogs and cats showed that Staphylococci and Streptococci were among the most prevalent bacteria in pets [35].

In this study, 31% and 17% of sampled animals were carriers of *Staphylococcus* spp. and *Streptococcus* spp. in their mouths, respectively. These bacteria were the second most common species isolated from dog and cat bite-wounds, respectively [36].

The prevalence of these two species was more common in non-purulent cellulitis/lymphangitis wounds than in abscesses or purulent wounds [36]. Staphylococci- and Streptococci-related cellulitis is more diffuse and typically less severe than that seen with *P. multocida* infections [21].

Although, *Pasteurella* species, in particular *P. multocida*, is one of the most common opportunistic pathogens found in the oral cavity of dogs and cats leading to human infection after bites or scratches [13], no data are available on the resistance patterns of *Pasteurella* originating from dogs and cats in Algeria.

The majority of the *Pasteurella* isolates in this analysis were susceptible to all drugs tested, with only eight (15.68%) isolates demonstrating resistance to at least one antibiotic, of which six belonged to dogs.

These results are consistent with published studies reported high resistance of this bacterium to some antimicrobials [37,38]. From a therapeutic point of view, erythromycin, penicillin, and ampicillin were the less effective antimicrobials in the treatment of bite wounds, while multidrug resistance profiles were reported in two isolates.

Typically, *Pasteurella* species are susceptible to penicillin, ampicillin, and amoxicillin-clavulanate which are the standard therapy of human *Pasteurella*-associated bite wound infection [14-39]. Tetracycline, in the case of penicillin allergy, is an effective alternative [14].

In a recent study, by Ujvári *et al*. [31], all *Pasteurella* strains isolated from cats and humans were susceptible to ampicillin and tetracycline.

It should also be noted that none of the 72 *P. multocida* isolated from infections of the respiratory tract in dogs and cats investigated in the BfT-Germ Vet program demonstrated resistance to any of the tested antimicrobials (penicillin, ampicillin, erythromycin, and tetracycline) [40].

A study conducted by Ferreira *et al*. [37] in Brazil investigated *P. multocida* isolates from cat gingiva and dog oropharynx for their antimicrobial susceptibility. This study showed low levels of resistance to penicillin (7.3%); however, lower susceptibilities to erythromycin in isolates from both dogs and cats were observed (21.27%). In same study, resistance to tetracycline was absent.

Although, resistance of *Pasteurella* to β-lactam antibiotics has been described, no resistance of *P. multocida* to tetracycline and doxycycline has been reported in humans or pet animals. Furthermore, resistance to tetracycline has been described in pig and ruminant strains [31]. Interestingly, we found a *P. multocida* strain resistant to tetracycline in dog.

At least nine tetracycline resistance genes (TET genes) have been detected in *Pasteurella* species [40]. Many of them are related to plasmids or transposons and can, therefore, be exchanged horizontally, not only within the *Pasteurellaceae* family, but also with other Gram-negative bacteria [40].

In addition, experiments on the molecular basis of antimicrobial resistance indicate that *Pasteurella* has obviously acquired a number of resistance genes from other Gram-negative or Gram-positive bacteria [40].

Erythromycin is a member of the antibiotic macrolide class; recently, macrolide resistance has emerged in *P. multocida* [31]. There are no descriptions of resistance to this antibiotic in ­companion animals [37], but lower susceptibility to erythromycin in *Pasteurella* isolates from both dogs and cats was common [38].

The exchange of resistance genes between different organisms in the oral cavity as well as between oral bacteria and bacteria from other environments has already been described [8].

## Conclusion

To the best of our knowledge, this study is the first in our country to detect zoonotic bacteria found in dogs and cats’ oral cavities. It demonstrates that stray dogs and cats in Algeria carry many opportunistic and/or potentially pathogenic bacteria in their mouths, including *Pasteurella* spp., leading to infection of bite wounds and scratches.

It was shown that dogs were more carriers of *Pasteurella* spp. than cats, some of them were multidrug-resistant, a risk to public health.

Amoxicillin-clavulanate and tetracycline were the most sensitive drugs against *Pasteurella* species. Thus, these antimicrobials may be sufficient in humans to treat infected bites caused by *Pasteurella*.

## Authors’ Contributions

KR, MNM, and KA conceived the study designed. KR performed the experiment and analyzed the data. KR and RK drafted and revised the manuscript. AA conducted the statistical analysis. All authors read and approved the final manuscript.
